# Defective Autophagy and Mitophagy in Aging and Alzheimer’s Disease

**DOI:** 10.3389/fnins.2020.612757

**Published:** 2021-01-08

**Authors:** Michael Tran, P. Hemachandra Reddy

**Affiliations:** ^1^Department of Internal Medicine, Texas Tech University Health Sciences Center, Lubbock, TX, United States; ^2^Neuroscience and Pharmacology, Texas Tech University Health Sciences Center, Lubbock, TX, United States; ^3^Neurology, Departments of School of Medicine, Texas Tech University Health Sciences Center, Lubbock, TX, United States; ^4^Public Health Department of Graduate School of Biomedical Sciences, Texas Tech University Health Sciences Center, Lubbock, TX, United States; ^5^Department of Speech, Language and Hearing Sciences, School Health Professions, Texas Tech University Health Sciences Center, Lubbock, TX, United States

**Keywords:** Alzheimer’s disease, mitochondria reactive oxygen species, mitophagy, autophagy, aging

## Abstract

Aging is the time-dependent process that all living organisms go through characterized by declining physiological function due to alterations in metabolic and molecular pathways. Many decades of research have been devoted to uncovering the cellular changes and progression of aging and have revealed that not all organisms with the same chronological age exhibit the same age-related declines in physiological function. In assessing biological age, factors such as epigenetic changes, telomere length, oxidative damage, and mitochondrial dysfunction in rescue mechanisms such as autophagy all play major roles. Recent studies have focused on autophagy dysfunction in aging, particularly on mitophagy due to its major role in energy generation and reactive oxidative species generation of mitochondria. Mitophagy has been implicated in playing a role in the pathogenesis of many age-related diseases, including Alzheimer’s disease (AD), Parkinson’s, Huntington’s, and amyotrophic lateral sclerosis. The purpose of our article is to highlight the mechanisms of autophagy and mitophagy and how defects in these pathways contribute to the physiological markers of aging and AD. This article also discusses how mitochondrial dysfunction, abnormal mitochondrial dynamics, impaired biogenesis, and defective mitophagy are related to aging and AD progression. This article highlights recent studies of amyloid beta and phosphorylated tau in relation to autophagy and mitophagy in AD.

## Introduction

**FIGURE 1:**
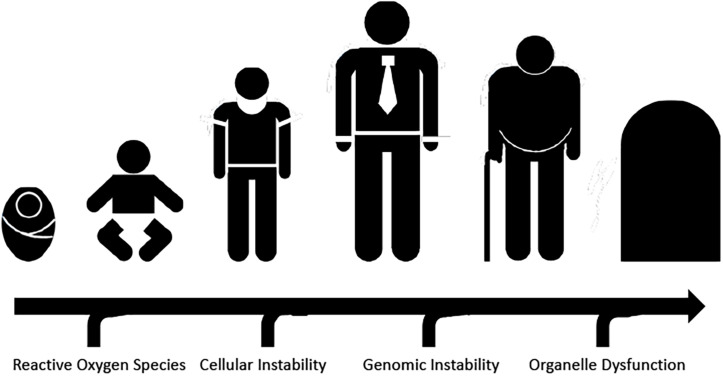
Normal physiological aging accelerated by factors leading to cellular dysfunction, including reactive oxygen species, organelle dysfunction, and genomic damage.

Aging is generally thought of as the time-dependent accumulation of cellular damage and decline in physiological function. Although many events can lead to cellular dysfunction, several factors have been identified as the defining characteristics of aging: genomic instability, telomere attrition, epigenetic alterations, loss of proteostasis, cellular senescence, stem cell exhaustion, altered intercellular communication, and mitochondrial dysfunction (Figure 1; Lopez-Otin et al., 2013). Accumulation of genetic damage is one of the hallmarks of aging, and the integrity of an organism’s genome is constantly being challenged (Moskalev et al., 2013). Exogenous threats include physical, chemical, and biological agents, whereas endogenous threats include DNA replication errors, spontaneous mutations, and alterations due to reactive oxygen species (ROS). To help protect from these threats, most organisms have evolved multiple forms of protective DNA mechanisms that collectively help minimize damage and maintain genomic stability.

One such mechanism is the existence of telomeres on the end of chromosomal DNA, which are DNA repeats that prevent erosion of coding segments of DNA during replication. Most DNA polymerases cannot completely replicate the terminal ends of linear DNA, with telomerase being the only specialized form capable of doing so. However, most mammalian cells do not express telomerase, which ultimately leads to the progressive loss of the protective factor of telomeres (Blackburn et al., 2006). Telomere shortening limits the proliferative capacity of all cells, and telomere depletion leads to cell senescence, which arrests cell cycle progress and halts replication. Epigenetic modifications are a form of genetic alteration that can be signs of aging. Epigenetic changes are involved in alterations in the methylation patterns of DNA, post-translational modification of histones, and chromatin remodeling, which can alter protein expression (Fraga and Esteller, 2007). Dysregulation of the epigenetic machinery is related to aging in invertebrates (Greer et al., 2010; Maegawa et al., 2010).

The purpose of this article is to critically examine the role of age-related factors in autophagy and mitophagy in AD pathogenesis. This article will also highlight how mitochondrial dysfunction, abnormal mitochondrial dynamics, and impaired biogenesis are related to aging and AD pathogenesis.

## Aging and Cellular Senescence

Aging is a major risk factor for a large number of neurodegenerative diseases, including Alzheimer’s disease (AD), Parkinson’s, and amyotrophic lateral sclerosis (ALS) (Reddy and Reddy, 2011). Aging occurs at different rates in different species, and interindividual variations exist within a species and in the different tissues of an individual. Aging is a progressive deterioration that leads to cell senescence and an increased risk of developing many diseases.

Cellular senescence is a series of cellular states after an initial growth arrest in which cells go through phenotypic alterations. Cellular senescence and aging are two distinct phenomena; aging is the progressive decline with time, whereas senescence occurs throughout the lifespan, including during embryogenesis. The number of senescent cells increases with age, but senescence also plays an important role during development. These events are directly associated with autophagy and mitophagy.

Hayflick and Moorhead first characterized senescence as the phenomenon of irreversible growth arrest linked to telomere attrition (Hayflick and Moorhead, 1961). Senescence helps guard against the continual replication of damaged cells and plays a role in embryonic development, wound healing, tissue repair, and aging. As cells age out, new cells must take their place, and eventually, the regenerative potential of tissue decreases as stem cells become exhausted. Hematopoiesis has been shown to decline with age resulting in reduced production of adaptive immune cells and anemia (Shaw et al., 2010). Furthermore, studies on aged mice have shown that hematopoietic stem cells go through decreased cell-cycle activity and division compared with younger mice (Rossi et al., 2007). These decreases in divisions have been correlated to the accumulation of DNA damage resulting in overexpression of cell-cycle inhibitory proteins such as P16^*INK*4*a*^, which is a known inducer of cell senescence.

As cells age, DNA damage accumulates not only in chromosomal DNA but also in mitochondrial DNA leading to mitochondrial dysfunction. Mitochondria play a key role in respiratory oxidation for ATP generation, and dysfunction leads to electron leakage and energy deficits (Green et al., 2011). Turnover of dysfunctional and damaged mitochondria is paramount to maintaining healthy cell populations, and impairment of turnover can lead to greater cell death. The effects of aging in mitochondrial dysfunction are discussed later in this paper. Although many factors lead to biological aging within an organism, one common trend is the accumulation of defects over time.

## Age-Related Factors in Defective Autophagy

Several lines of research revealed that multiple age-related factors are implicated in defective autophagy (Cheon et al., 2019). In the last decade, several groups studied autophagy and mitophagy in aging and age-related diseases of different species such as flies, worms, humans, rats, and mice. Studies have also been done on rodent models of human disease and postmortem brains of healthy humans and humans with neurodegenerative disorders (Palikaras et al., 2015, 2018; Schiavi et al., 2015; Harper et al., 2018; Reddy et al., 2018; Manczak et al., 2018; Andreux et al., 2019; Castellazzi et al., 2019; Fang et al., 2019a,b; Hou et al., 2019; Martín-Maestro et al., 2019; Reddy and Oliver, 2019; Oliver and Reddy, 2019a,b; Wang et al., 2019; Li et al., 2020; Lou et al., 2020; Chen et al., 2020; Bakula and Scheibye-Knudsen, 2020; Liang and Gustafsson, 2020; Varghese et al., 2020; Luo et al., 2020; Markaki and Tavernarakis, 2020; Babbar et al., 2020; Aman et al., 2020; Cai and Jeong, 2020; Pakpian et al., 2020; Oh et al., 2020; Yang et al., 2020; Han et al., 2020; Pradeepkiran and Reddy, 2020). These articles (original and high impact review articles) have provided a large body of useful information about autophagy and mitophagy in different species of vertebrates and non-vertebrates.

Age-related factors, including oxidative stress, DNA damage, and telomere shortening, are involved in defective autophagy (Cheon et al., 2019) in both vertebrates and non-vertebrates. We briefly discuss these factors below:

### Oxidative Stress and Defective Autophagy

Mitochondria are the powerhouses of cells, providing energy for several cellular functions, including intracellular calcium regulation, ATP production, the release of proteins that activate the caspase family of proteases, and the alteration of the reduction–oxidation potential of cells and free radical scavenging. Cellular aging induces mitochondrial ROS production and disrupts the electron transport chain (ETC). Disruption of the ETC has been recognized as an early feature of apoptotic cell death. The ETC involves the reduction of hydrogen peroxide (H2O2) to H2O and O2 by superoxide dismutase, catalase, peroxidase, and glutathione accepting electrons donated by NADH and FADH2, which yields the energy for the generation of ATP from adenosine diphosphate and inorganic phosphate (Reddy, 2006, 2008). Mitochondrial superoxide radical (O•- 2) production occurs primarily at discrete points in the ETC at complexes 1 and 3 and in components of tricarboxylic acid, including α-ketoglutarate dehydrogenase. In addition, mitochondrial O•- 2 are generated in the outer mitochondrial membrane. Monoamine oxidase, localized on the outer mitochondrial membrane, catalyzes the oxidative deamination of primary aromatic amines (Reddy, 2006). This deamination produces a large amount of H2O2 that contributes to an increase in the steady-state concentrations of ROS within the mitochondrial matrix and the cytosol. These released H2O2 and O•- 2 are carried to the cytoplasm via voltage-dependent anion channels and ultimately lead to the oxidation of cytoplasmic proteins.

The age-related chronic exposure cells to ROS can result in oxidative damage to mitochondrial proteins, cellular proteins, lipids, and nucleic acids, whereas the acute exposure to ROS can inactivate the tricarboxylic acid-cycle aconitase and the iron–sulfur centers of ETC at complexes 1, 2, and 3, resulting in a shutdown of mitochondrial energy production (Reddy, 2008). Therefore, mitochondria undergo morphological and functional changes with age, including declines in ETC function, mitochondrial integrity, and mitochondrial quality, which results in impairments of cellular energy production and activity.

Autophagy plays a key role in the clearance of damaged cellular organelles, including mitochondria. However, age-related impairments of autophagy lead to the accumulation of abnormal mitochondria, which increases oxidative stress. Based on these studies, it is proposed that mitochondria-targeted antioxidants, such as MitoQ, and SS31 can be potential drugs that reduce free radicals, maintain mitochondrial quality and function, boost autophagy and mitophagy, and clear damaged mitochondria from cells.

### DNA Damage and Defective Autophagy

Oxidative stress, which is caused by an imbalance between the production of free radicals and the presence of endogenous antioxidants within a cell, is the major cause of damage to DNA (Reddy, 2006, 2008). During aging and age-related conditions, free radicals’ increased production occurs, and this increase of free radicals damages both nuclear and mitochondrial DNA (Reddy and Beal, 2008; Oliver and Reddy, 2019a,b). DNA damage is distinctly different from the germline mutation, but both lead to errors in DNA. DNA damage is an abnormality in the chemical structure of DNA, whereas a mutation is a change in the sequence of standard base pairs. Damage to DNA can cause changes in the structure of the genetic material and prevents and/or alters the replication of DNA (Cheon et al., 2019).

Age-dependent DNA damage plays a large role in defective autophagy. The generation of free radicals can occur after several cellular insults, including ultraviolet irradiation damage of DNA and redox-cycling of quinones (Cheon et al., 2019). Both mitochondrial damage and nuclear DNA damage occur in an age-dependent manner. Somatic mitochondrial changes, including single nucleotide changes and large deletions, have been extensively reported in both vertebrates and non-vertebrates (Reddy and Beal, 2005; Oliver and Reddy, 2019a,b). DNA base-pair repair is defective and increased in an age-dependent manner. As mentioned earlier, mitochondrial DNA is more vulnerable to ROS than nuclear DNA because of its lack of protective shields—histones (Reddy and Beal, 2005; Oliver and Reddy, 2019a,b). Mutations of mitochondrial DNA (mtDNA) are usually due to replication errors by mtDNA polymerase and point mutations/deletions that spontaneously accumulate during aging. Several DNA repair events are activated in response to damaged DNA, including homologous recombination repair, non-homologous end joining, mismatch repair base excision repair, and nucleotide excision repair (Reddy and Beal, 2005; Oliver and Reddy, 2019a,b).

Previous studies support that the base excision repair is mainly involved in the repair of oxidative mtDNA modification and mitigates mitochondrial impairment. Mismatch repair-dependent autophagy requires Bcl-2-interacting protein 3 in a mammalian target of rapamycin (mTOR)-dependent manner (Cheon et al., 2019). Decreased ability to repair DNA and consequent accumulation of DNA damage may contribute to cellular senescence. Also, mutations in nuclear and mitochondrial genes caused by impaired DNA repair have been associated with aging.

Overall, DNA damage plays a significant role in defective autophagy and is directly associated with aging and age-related diseases such as AD, Parkinson’s, and ALS.

### Telomere Shortening and Defective Autophagy

What are telomeres?—these are specific DNA–protein structures found at both ends of each chromosome that protect the genome from nucleolytic degradation, unnecessary recombination or repair, and inter-chromosomal fusion. Telomeres, therefore, play a vital role in preserving the information in our genome (Shammas, 2011). As a normal cellular process, a small portion of telomeric DNA is lost with each cell division. Telomeres become shorter with aging, influenced by environmental factors and specific genetic defects in the underlying telomere mechanisms. When telomere length reaches a critical limit, the cell undergoes senescence and/or apoptosis. Telomere length, therefore, serves as a biological clock to determine the lifespan of a cell and an organism.

Telomerase is a reverse transcriptase enzyme complex capable of adding DNA sequence repeats (TTAGGG) to the 3′ end of DNA strands in the telomere regions at the ends of eukaryotic chromosomes (Harris and Cheng, Nephron 2017). Telomerase contains two major components in its transcriptase ribonucleoprotein complex, the RNA-directed DNA polymerase, TerT, and the RNA template, TerC, which together prevent telomere shortening by adding telomeric DNA repeats to chromosome ends. However, telomeric DNA repeats become defective in aging and age-related conditions.

A gradual loss of telomeric DNA leads to defective autophagy cellular events. Earlier studies have demonstrated that shortened telomeres are associated with autophagy (Aoki et al., 2007; Ali et al., 2016; Nassour et al., 2019). In cells with shortened telomeres, autophagy-related proteins and cytoplasmic vacuoles were increased (Nassour et al., 2019). Telomeric 3′ DNA oligonucleotides can induce autophagosomes and inhibit mTOR signaling in malignant glioma cells (Aoki et al., 2007). In addition, in multiple cell lines such as HEK 293T, HepG2, and U-2 OS, TERT binds to mTORC1 kinase and suppresses its activity, inducing autophagy. On the other hand, TERT knockdown increases the components of mTORC1, resulting in autophagy impairment under basal starvation conditions (Ali et al., 2016).

Overall, the shortening of telomeres impacts autophagy in aging and age-related diseases. Based on these studies, it has been proposed that autophagy and mitophagy enhancers are potential therapeutic targets.

## Autophagy

Autophagy is the lysosome-mediated self-degradative process that plays a major role in nutrient balancing and housekeeping by selectively degrading dysfunctional organelles and proteins (Deter and De Duve, 1967; Glick et al., 2010; Parzych and Klionsky, 2014; Pradeepkiran and Reddy, 2020). Autophagy is carried out by a class of proteins called autophagy-related proteins (Atg), which were discovered in yeast cells. Atg proteins have been found throughout many different types of organisms, including mammalian cells, with several analogs within mammalian cells being identified with similar mechanisms as Atg proteins within yeast cells. When the mechanisms underlying autophagy are disrupted, cells become more prone to accumulating defects leading to reduced cell viability. If the autophagic pathways are not rescued, the accumulation of cellular debris can occur and lead to an acceleration of physiological aging (Rubinsztein et al., 2011). Conversely, normal aging associated with accumulations of DNA damage due to oxidative damage can lead to dysregulation of the autophagy machinery (Vellai, 2009). This leads to a positive-feedback loop in which normal cellular damage experienced in aging leads to a decreased ability for cells to protect themselves from further insult compromising cellular viability.

**FIGURE 2:**
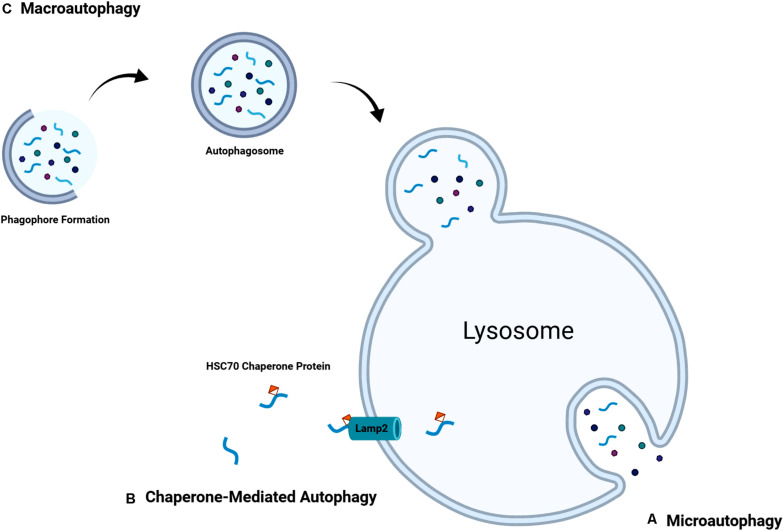
Overview of various forms of autophagy including, **(A)** microautophagy in which lysosomal invaginations directly take up cellular substrate, **(B)** chaperone-mediated autophagy in which chaperone proteins target specific proteins to lysosomal membrane proteins, and **(C)** macroautophagy in which phagophore formation around cellular substrate occurs and is trafficked to the lysosome where it will fuse.

Autophagy can work in both a non-selective or selective manner, which targets organelles such as peroxisomes, mitochondria, or portions of the endoplasmic reticulum for degradation. Three modes of autophagy have been characterized: macro-autophagy, micro-autophagy, and chaperone-mediated autophagy (Figure 2). Although each mode of autophagy is mechanically distinct, all three ultimately lead to the delivery of cellular cargo to the lysosome or vacuole for degradation and recycling (Parzych and Klionsky, 2014). Many factors can induce autophagy within a cell, which often center on mitochondria. Release of cytochrome c, increased ROS production, the opening of mitochondrial permeability transition pore (mPTP), and oxidative damage all lead to selective autophagy of damaged mitochondria deemed mitophagy (Quinsay et al., 2010). When mitophagic function is overwhelmed, autophagy can be initiated on a grander scale leading to cell death and apoptosis.

As discussed earlier, autophagy is defective in aging and age-related diseases, mainly due to reduced clearance of subcellular organelles/proteins, such as mitochondria, endoplasmic reticulum, and other cellular debris. We discuss the detailed events of autophagy later.

### Microautophagy

Microautophagy is a process in which a lysosome or vacuole will engulf cellular material directly in response to states of starvation (Mijaljica et al., 2011; Oku and Sakai, 2018; Yoo and Jung, 2018). The mechanisms that regulate microautophagy are poorly understood, with most microautophagy research being performed primarily in a yeast cell. These studies focused primarily on the mechanism by which lysosomes engulf peroxisomes by altering the environmental carbon source from methanol to glucose (Sienko et al., 2020). Microautophagy can be carried out both in a non-selective and selective process that targets proteins and organelles. In the non-selective form of microautophagy, the mechanism of absorption is based on the size of what is being engulfed. For smaller cytosolic contents such as proteins, tubular invaginations form in the vacuolar membrane, which then pinch off into autophagic vesicles. The invaginations are formed through the action of a GTPase and can occur without the normal proteins of vacuole fusion such as SNAP receptors and N-ethylmaleimide sensitive factor.

Larger structures like organelles cannot fit in these invaginations, so the vacuole will form finger-like projections that can engulf cellular contents and fuse to absorb the contents into the vacuole. This process is partially dependent on the target of rapamycin (TOR) and exit from the rapamycin-induced growth arrest (EGO) complex. The TOR complex is also related to macroautophagy, and the intersection of these processes is counterbalanced in such a way that excessive autophagy does not occur (Oku and Sakai, 2018). Utilizing yeast mutants, studies have shown that some Atg genes that are involved in macroautophagy also play as key enzymes in microautophagy (Dunn et al., 2012). Micropexophagy, the selective autophagy of peroxisomes, is carried out in a series of steps, including initiation, target recognition, peroxisome sequestration, and terminal vacuole enclosure, and it has been shown that all of these steps are mediated by a series of Atg proteins. It has also been shown that non-selective microautophagy and other selective autophagy are reliant on Atg proteins as well (Sienko et al., 2020). The exact role Atg proteins plays in microautophagy, and the regulating mechanisms surrounding microautophagy are poorly understood and warrant further study. One key challenge in understanding these processes is how it is being studied; microautophagy induced *in vitro* does not accurately represent physiological states *in vivo*.

### Chaperone-Mediated Autophagy

Chaperone-mediated autophagy (CMA) is a form of autophagy in which specific proteins are targeted by chaperone proteins and trafficked to lysosomes where they directly enter through the membrane (Majeski and Dice, 2004; Dice, 2007; Li et al., 2012). CMA has only been found in higher eukarya and is an important mechanism for the maintenance and regular turnover of cellular proteins to help ensure optimal function. Although CMA is also used in times of energy deficiency, activation of this pathway is slower than macroautophagy and may play a more important role in regulating the amounts of specific proteins in metabolic pathways. The constitutive chaperone, heat shock-cognate protein of 70 kDa (hsc70), targets a pentameric motif within proteins for autophagy. In some cases, the motif is not accessible by hsc70 until the protein becomes unfolded due to degradation or if post-translational modification by other proteins alters the charge state of a pentamer to the appropriate motif. Once the hsc70-protein dimer arrives at the lysosome, it interfaces with lysosome-associated membrane protein type 2A (LAMP-2A), forming a complex with other lysosomal membrane proteins to allow for the translocation of the substrate directly into the lysosomal lumen for degradation. The maximal rate of CMA is primarily dependent on the amount of LAMP-2A within the lysosomal membrane (Li et al., 2012). LAMP-2A transcription is increased in times of oxidative stress and decreased in times of prolonged starvation.

Malfunctions in the CMA pathway play a key role in the pathogenesis of many human disorders, including some neurodegenerative diseases (Dice, 2007). Decreases in the CMA pathway compromise a cell’s ability to properly remove deleterious proteins leading to accumulation within cells. Such accumulations alter proteostasis and can lead to the deposition of protein aggregates leading to neuronal demise (Majeski and Dice, 2004). It has been observed that in normal physiological aging, CMA decreases. Age-related changes in CMA activity have been linked to alterations in the lipid composition of lysosomal membranes, which threatens the integrity of LAMP-2A proteins (Cuervo and Wong, 2014). CMA function is imperative in maintaining protein integrity and ensuring proper cellular function.

### Macroautophagy

Macro-autophagy is the most studied and most understood mode of autophagy. The defining mechanism of macro-autophagy is the *de novo* formation of a double-membrane vesicle termed “autophagosomes,” which engulfs cellular debris. Macro-autophagy follows five steps for breakdown: (1) induction of the isolation membrane, (2) elongation of membrane, (3) closure and autophagosome formation, (4) autophagosome–lysosome fusion, and (5) lysosomal degradation. In yeast, the formation of autophagosomes is initiated at the phagophore assembly site, which is a single site adjacent to the vacuole (Glick et al., 2010). In eukaryotes, the formation of autophagosomes is initiated at multiple sites throughout the endoplasmic reticulum called omegasomes. The initial formation of the double-membrane is termed the phagophore, which rapidly begins to expand into the spherical autophagosome. The autophagosome will bend and engulf its target within its double-membrane and then be translocated to the lysosome or vacuole. Once at the target site, the autophagosome will fuse with the lysosome, at which point it becomes the autolysosome. The acidic contents of the lysosomal lumen then break down the autophagosomal membrane as well as its contents, and the breakdown products are exported back into the cellular cytoplasm for reuse in biosynthetic pathways. In mammals, autophagosomes can integrate into the endocytic pathway and fuse with endosomes, which are subsequently broken down together (Deter and De Duve, 1967).

The mechanisms by which macroautophagy is regulated were first studied in yeast, and many autophagy-related genes (Atg) were identified. Many of the Atg proteins are found in other eukaryotic organisms, including mammals, but some of the Atg proteins have homologs, such as the Unc-51-like kinase family, which serves the same role as yeast Atg13 (Parzych and Klionsky, 2014). The mammalian system of macroautophagy is more complex than yeast systems and includes many additional regulatory proteins. Both yeast and mammalian autophagosomes require proteins that regulate autophagosome nucleation and elongation.

Several stressors trigger macroautophagy, such as nutrient deficiency, insulin concentrations, endoplasmic reticulum stress, and energy levels. Due to the role macroautophagy plays in cellular recycling, autophagosome formation is tightly regulated by two major pathways that are sensitive to carbon and nitrogen balance. The cAMP-dependent protein kinase A (PKA) pathway regulates macroautophagy by sensing carbon balance within both yeast and mammalian cells. PKA activation by high cAMP concentrations signals a nutrient-rich state within the cell and will inhibit further cellular recycling by autophagy. The mammalian target of rapamycin complex 1 (MTORC1) is a key protein in macroautophagy that is sensitive to amino-acid levels within a cell, which in turn is an indicator of the nitrogen balance (Kiffin et al., 2007). Some studies have shown that PKA interacts with MTORC1 and can phosphorylate and activate MTORC1, suggesting that these pathways are linked. In mammalian cells, AMP-activated protein kinase (AMPK) is a substrate of PKA and is a major energy-sensing kinase that responds to cellular AMP levels, which is a strong indicator of the energy level within a cell. Yeast has a similar mechanism in which macroautophagy is regulated by energy-sensing using the Snf1, which works similarly to AMPK. Endoplasmic reticulum stress can also precipitate autophagosome formation. Calmodulin-dependent protein kinase 2, beta (CaMKKβ) activation leads to increased cytosolic Ca^2+^ concentrations, which can induce AMPK and subsequently macroautophagy. The endoplasmic reticulum can also detect unfolded proteins, which will induce macroautophagy through similar mechanisms. As macroautophagy is the most well-studied of autophagic pathways, we will use the term autophagy to describe it hereafter.

### Proteins of Autophagy

Due to the importance of autophagy in maintaining cellular integrity and health, many proteins are involved in the induction and maintenance of these pathways. Although not an exhaustive list, we will briefly discuss some of the major proteins of autophagy in the following sections.

#### Mammalian Target of Rapamycin

Mammalian Target of Rapamycin is a complex of 2 protein kinases consisting of MTOR1 and MTOR2 that is sensitive to cellular nutrition levels and plays a role in regulating cell growth and survival via autophagy (Hale et al., 2012; Lett, 2016). MTOR is integrated into many cell survival pathways and utilizes nutrition levels to modulate cell growth. In nutrient-rich conditions, MTOR is activated, leading to the phosphorylation of key autophagy enzyme Unc-51-like kinase-1 (ULK1). Phosphorylation of ULK1 suppresses protein activity and prevents phagosome formation.

#### Unc-51-Like Kinase-1/ATG13

Unc-51 like kinase-1 is a protein associated with MTOR that will dissociate in response to nutrient-poor cellular conditions. ULK1 then acts to phosphorylate both ATG13 and RB1-inducible coiled-coil 1, which are proteins required to form phagosomes around cellular content (Khang et al., 2011). ULK1 also acts on a multitude of proteins involved in autophagy progression and regulation, including Beclin1 and Ambra1.

#### Beclin1 and Ambra1

Beclin1 is a key protein of autophagy activated by ULK1. Once activated, Beclin1 promotes the formation of the Vps34 complex, which consists of BCL1, Vps34/CIII PI3K, and Vps15 (Lett, 2016). The Vps34 complex is a key regulator of autophagy initiation and progression. Beclin1 contains three structural domains, a Bcl-2 homology 3 (BH3) domain, a central coiled-coil domain, and an evolutionarily conserved domain. Under normal cellular conditions, the BH3 domain interacts with Bcl-2 to inhibit autophagy. The coiled-coil domain interacts with multiple proteins that promote activation of autophagy, including Ambra1, UV radiation resistance association gene, and Atg14L (Khang et al., 2011). The evolutionarily conserved domain allows Beclin1 to modulate autophagy and inhibit tumorigenesis. As previously noted, Ambra1 is an essential activator of the Beclin1-dependent pathway of autophagy, but it also promotes stabilization of ULK1 and kinase activity (Maria Fimia et al., 2007).

#### Phagosome Elongation

Phagosome completion around targeted cytosolic content is accomplished by a series of ATG genes primarily through 2 ubiquitin-like systems. ATG7 is activated in an ATP-dependent manner, which then activates ATG12. ATG12 then is complexed to ATG5 by ATG10, an E2-like enzyme, and forms the first complex, ATG5-ATG12-ATG12L1. This complex works to elongate the phagophore. The second system starts with MAP1LC3, the mammalian ATG8 homolog, and ATG4B. ATG4B cleaves LC3 into LC3-I, which is conjugated to phosphatidylethanolamine via ATG3 and ATG7 forming LC3II. LC3II is integrated into the nascent phagosome membrane and acts as a marker that facilitates phagophore fusion with lysosomes (Hale et al., 2012). Together, these two systems help complete phagosome formation and target it to the lysosome for degradation.

## Selective Autophagy

Autophagy in response to nutrient imbalances usually occurs as a non-specific process, but it can also be conducted in a highly specific manner for cell maintenance by targeting peroxisomes, mitochondria, and other organelles (He and Klionsky, 2010). Selective autophagy plays a role in destroying malignant cells, damaged organelles, invasive pathogens, protein aggregates, and excess peroxisomes. In selective autophagy, autophagosomes target specific cargo using the Atg-8 family proteins on the isolation membrane (Mehrpour et al., 2010). CMA also only works as a selective process, utilizing Hsc-70 chaperone proteins to traffic targeted proteins to lysosomal receptors for recycling. In mammalian cells, Atg8 analogs such as LC3 and GABARAP help to selectively sequester target substrate within autophagosomes utilizing cargo-specific receptors (Majeski and Dice, 2004; Dice, 2007; Li et al., 2012). LC3-interaction regions (LIR) have been shown to not only play a role in autophagy but also recruit other autophagosomal proteins. Ubiquitin is a well-known marker targeting proteins for degradation, and it is also used to mark cellular material for autophagy. Other pathways exist for specific autophagy independent of ubiquitin as well. Many selective autophagy pathways have been discovered and named according to the cellular target, such as mitochondria (mitophagy), ribosomes (ribophagy), endoplasmic reticulum (reticulophagy), peroxisomes (pexophagy), and many other organelle-specific mechanisms. Although selective autophagy ultimately utilizes many of the same mechanisms underlying non-selective autophagy, certain proteins and factors are associated with each organelle that will induct the autophagy machinery (He and Klionsky, 2010; Zaffagnini and Martens, 2016). Although all forms of specific autophagy play an important role in cell maintenance and health, we focus on mitophagy in this paper.

### Mitophagy

As previously discussed, mitophagy is the selective autophagy of mitochondria and has been a focus of research in recent years for its potential role in many diseases (Lynch-Day and Klionsky, 2010; Youle and Narendra, 2011; Pradeepkiran and Reddy, 2020). Mechanisms underlying mitophagy are best documented in yeast studies, and several proteins are essential for mitophagy (Ding and Yin, 2012). One such protein is Uth1, a SUN-domain protein essential for mitophagy in yeast (Ashrafi and Schwarz, 2013). Ancient ubiquitous protein, a phosphatase 2C, plays a role in facilitating mitophagy in yeast in a stationary phase (Tal et al., 2007; Youle and Narendra, 2011). Ancient ubiquitous protein is not required for non-specific autophagy. Although Atgs are utilized in all autophagy pathways, a few have been shown to play a role in the selective uptake of mitochondria. In two studies, it has been shown that Atg 32 is a mitochondrial receptor capable of inducing mitophagy in yeast. Atg32 can bind to Atg11, which acts as an adaptor to Atg8, which is thought to signal mitochondria absorption into autophagosomes (Kanki et al., 2009; Okamoto et al., 2009).

Although these proteins are essential in yeast for mitophagy, no homologs have been found in mammalian cells with their own pathways for initiating mitophagy. Although mitochondria play a pivotal role in protecting cells from oxidative stress, they themselves are not immune to the destructive effects of reactive species. Proteins involved in mitophagy that are responsible for the upkeep of healthy mitochondrial populations are encoded by nuclear DNA and so are subject to the mutative effects of oxygen species (Sun et al., 2016; D’Amico et al., 2019). Furthermore, post-translational modifications observed in aging have been associated with decreased expression of mitophagy proteins, which accelerates the aging process. Studies have demonstrated that Pumilio2 (Pumilio homolog 2 is an RNA-binding protein) regulates synaptic plasticity via translational repression of synaptic receptors and is activated in aging leading to suppression of mitochondrial fission factor. This disrupts mitochondrial dynamics, ultimately leading to decreased mitochondrial fission and inhibiting mitophagy of abnormal mitochondria. Both mitochondrial function and the ability of a cell to conduct mitophagy are expected to decrease in normal physiological aging; these effects are exacerbated in aging-related diseases such as AD, which accelerates the decay of mitochondrial function. Due to the essential role of mitophagy in maintaining cellular health, several mechanisms for its regulation exist. Although not an exhaustive list, the two most predominant pathways are discussed in the next sections.

### PTEN-Induced Putative Kinase 1/Parkin Pathway

**FIGURE 3:**
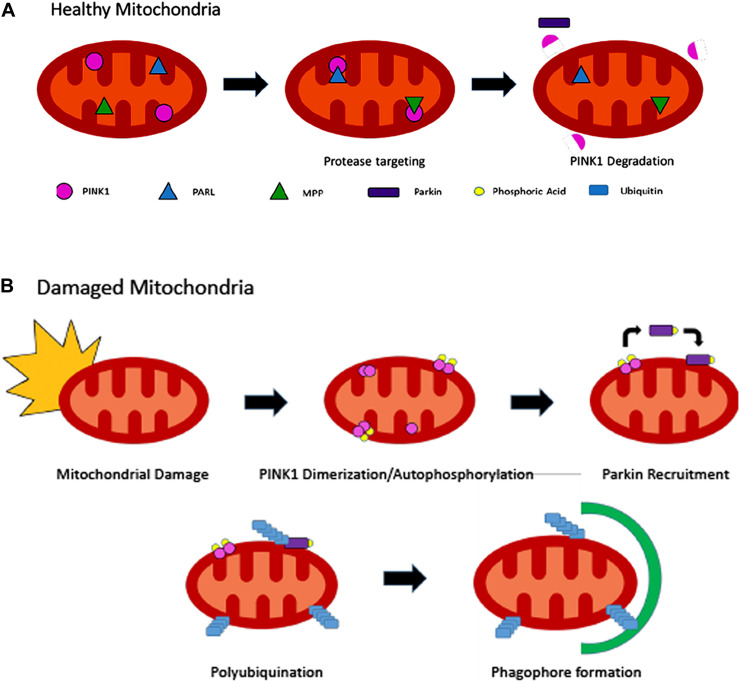
Mechanism by which PINK1/Parkin pathway for autophagy occurs in healthy **(A)** and damaged mitochondria **(B)**.

One of the most well understood and important regulators of mitophagy is the PINK1/Parkin pathway (Figure 3). PTEN-induced putative kinase 1 (PINK1) is a serine/threonine kinase localized in the inner mitochondrial membrane. In healthy mitochondria, PINK1 is constantly degraded by mitochondrial proteins, including matrix processing peptidases and presenilin-associated rhomboid-like (Youle and Narendra, 2011; Ding and Yin, 2012; Ashrafi and Schwarz, 2013). Fragmented PINK1 is then translocated to the cytosol and further degraded by other proteases. In damaged mitochondria, the mitochondrial membrane becomes depolarized, deactivating matrix processing peptidases and presenilin-associated rhomboid-like. PINK1 can then auto-phosphorylate, leading to activation and accumulation on the outer mitochondrial membrane, where it then can recruit cytosolic Parkin (Pickrell and Youle, 2015). Parkin, an E3 ubiquitin ligase, is phosphorylated by PINK1 and subsequently translocated into the mitochondrial membrane (Ashrafi and Schwarz, 2013). Once phosphorylated, Parkin enters the mitochondria, where it is believed to play a role in the ubiquitylation of mitochondrial proteins and substrate, marking the mitochondria for autophagy. Once protein Parkin works on, mitofusin 1, becomes activated, leading to mitochondrial fission, allowing for easier mitophagy (Pickrell and Youle, 2015). Ultimately, phosphorylation of Parkin by PINK1 leads to the initial induction of mitophagy in damaged or defective mitochondria.

### BNIP3/NIX Pathway

BCL2/adenovirus E1B 19 kDa protein-interacting protein 3 and NIX are both transmembrane proteins found in the outer mitochondrial membrane that share a similar structure and play a role in mitophagy (Zhang and Ney, 2009; Ney, 2015). These proteins were initially found to play a role in the selective exocytosis of mitochondria from nascent red blood cells, and their presence is required for the final maturation of RBCs. BNIP3 contains a BH3 domain as well as a carboxyl-terminal transmembrane domain, which works as a pro-apoptotic factor. The transmembrane of the domain of BNIP3 is required for mitochondrial targeting and subsequent pro-apoptotic activity (Ney, 2015). Expression of BNIP3 and NIX has been shown to increase in hypoxic cell conditions leading to cytochrome C release, membrane depolarization, and mitochondrial swelling. Nix has also been shown to directly interact with LC3 and GABARAP, which are inducers of the general autophagic machinery (Ding et al., 2010). Although the exact role of BNIP3 and NIX in the induction of mitophagy is not well understood, several potential mechanisms have been proposed. The role of these proteins in membrane depolarization has been shown to play a role in the induction of general autophagy mechanics, and Nix may play a role in ROS generation, which leads to the recruitment of markers for autophagy. A second theory is that BNIP3 and NIX competitively bind BCL2, liberating Beclin-1, which can then activate autophagy. The last theory is that BNIP3 inhibits Rheb, an upstream target of the mTOR, thereby inducing autophagy (Ney, 2015). Most studies suggest that BNIP3 and NIX do not act through a singular pathway and that they may work through several pathways to promote mitophagy and cell death. Although other pathways are theorized to play a role in mitochondrial autophagy, they are not well understood and believed to work in mechanisms similar to those outlined earlier.

## Alzheimer’s Disease

Alzheimer’s disease is an insidious neurodegenerative disease characterized by the presence of senile plaques composed of amyloid-beta (Aβ) peptides and the formation and accumulation of hyper-phosphorylated tau into neurofibrillary tangles in the brain (Selkoe, 2010; Hyman et al., 2012; Reiss et al., 2018; Shefa et al., 2019). Although the exact pathogenesis of AD is not well understood, it is believed that both Aβ and neurofibrillary tangles play a major role in the progression and symptoms present in AD. AD is one of the most common forms of dementia worldwide and is believed to be the sixth leading cause of death in the United States (Reiss et al., 2018). Increases in life expectancy have greatly increased the number of cases of dementia, driving more research into the underlying causes and progression of aging and AD. The defining features of AD include cognitive impairment due to synaptic dysfunction, increased confusion, and issues with short-term memory loss, which progressively worsens (Reddy et al., 2017). Most cases of AD are sporadic and occur late in life, but genetic components are found in approximately 2% of cases and are termed “familial” AD with symptoms manifesting as early as 30–40 years of age. Most cases of familial AD are associated with genetic alterations in genes coding for proteins related to the processing of amyloid precursor protein or amyloid precursor protein itself. In particular, mutations in the presenilin 1 (PS1), a core component of γ-secretase, which plays a role in APP processing, is a major risk factor for developing early-onset AD (Goiran et al., 2018). Alterations in the processing proteins of APP leads to an increased amount of Aβ-42, which is believed to play a role in AD pathogenesis. Diagnosis of AD is through cognitive testing supported with imaging techniques such as magnetic resonance imaging. However, due to the normal, expected cognitive decline with age, it can be difficult to diagnose AD in the early stages of the disease.

### Amyloid Beta

In AD, accumulation of Aβ into oligomers and fibrils is implicated as one of the early events in AD development and progression (Reddy, 2006; Reddy and Oliver, 2019; Murphy and Levine, 2010; Hamley, 2012). Aβ plaques accumulate in the hippocampus, amygdala, and associated neocortex, all of which play roles in memory formation. Aβ is generated as the breakdown of amyloid precursor protein (APP) by cleavage via secratases. Although the exact role of APP is not well understood, the breakdown products, including Aβ, play a role in AD (Zhang et al., 2011; Lauritzen et al., 2019). APP is processed via two different pathways, including the non-amyloidogenic pathway in which APP is cleaved by α-secretase to produce sAPPα, which is believed to play a role in neuronal survival and is further processed by γ-secretases to produce p83. The second pathway, termed the amyloidogenic pathway, involves APP processing by β-secretases and subsequent processing by γ-secretases into Aβ. Although the first pathway produces benign breakdown products of APP, the formation of Aβ as well as the intermediates of the amyloidogenic pathway are associated with AD pathogenesis. Although many lines of research have demonstrated an association between Aβ and AD, more recent lines of research have suggested that some of the other breakdown intermediates of APP, namely C9, may also play a role in AD (Laurtizen et al., 2016). Cells with impaired lysosomal function show increased levels of C99, which has been associated with AD. Furthermore, in studies with mice with increased expression of C99, neuronal populations displayed significantly reduced long-term potentiation, suggesting that C99 may play a role in AD progression. Studies with transgenic mice have demonstrated that C99 upregulation can lead to AD pathology even in the absence of Aβ (Laurtizen et al., 2016).

Overall, the accumulation of Aβ leads to mitochondrial dysfunction, synaptic damage, and defective autophagy within neuronal cells.

### Abnormal Interactions of Amyloid Beta With Drp1 and Defective Mitophagy

Abnormal interactions of amyloid beta and phosphorylated tau with mitochondrial and other cellular proteins have been reported in AD (Manczak et al., 2011; Manczak and Reddy, 2012a,b; Reddy and Oliver, 2019).

Reddy Lab (Manczak et al., 2011) investigated the molecular links between increased mitochondrial fission protein Drp1 and Aβ using co-immunoprecipitation and colocalization studies. Utilizing postmortem AD brains and brain tissues from APP mice and Drp1 immunoprecipitation/immunoblotting analysis of Aβ antibodies 6E10 and A11 revealed that Drp1 interacts with Aβ monomers and oligomers in AD patients and APP mice. These abnormal interactions are increased with disease progression. Their colocalization studies using Drp1 and the Aβ antibodies revealed the colocalization of Drp1 and Aβ (Manczak et al., 2011). These findings suggest that increased production of Aβ and the interaction of Aβ with Drp1 are crucial factors in mitochondrial fragmentation, abnormal mitochondrial dynamics, and synaptic damage in AD.

### Amyloid Beta Interaction With Voltage-Dependent Anion Channel 1 and Defective Mitophagy

To determine the role of mitochondrial outer membrane protein, voltage-dependent anion channel 1 protein (VDAC1) in AD, the Reddy group (Manczak and Reddy, 2012b) used brain specimens from AD patients and control subjects and 6-, 12- and 24-month-old Aβ precursor protein transgenic mice to assess VDAC1 protein levels. Furthermore, they also studied the interaction between VDAC1 and Aβ (monomers and oligomers) using cortical tissues from AD patients, control subjects, APP, APP/PS1, and 3XTg.AD mice. They also studied age- and VDAC1-linked, mutant APP/Aβ-induced mitochondrial dysfunction in APP and non-transgenic wild-type (WT) mice. Progressively increasing levels of VDAC1 in the cortical tissues from the brains of patients with AD were observed relative to control subjects, and significantly increased levels of VDAC1 were found in the cerebral cortices of 6-, 12- and 24-month-old APP transgenic mice relative to the age-matched control WT mice. Co-immunoprecipitation and co-labeling analysis of postmortem AD brains and brain tissue from APP transgenic mice revealed that VDAC1 interacted with Aβ in the brains of AD patients and APP, APP/PS1, and 3XTg.AD mice. They found progressively increased mitochondrial dysfunction in APP mice relative to control WT mice. Based on these observations, they concluded that VDAC1 interacts with Aβ and may in turn block mitochondrial pores leading to mitochondrial dysfunction in AD pathogenesis.

Based on these observations, they propose that reduced levels of VDAC1, Aβ, and phosphorylated tau may reduce the abnormal interaction between VDAC1 and APP, VDAC1 and Aβ. Reduced levels of VDAC1 and Aβ may maintain normal mitochondrial pore opening and pore closure, ultimately leading to normal mitochondrial function, allowing mitochondria to supply ATP to nerve terminals and boosting synaptic and cognitive function in AD (Manczak and Reddy, 2012b).

### Phosphorylated Tau

Tau proteins normally play a role in the assembly and stabilization of microtubules and other cytoskeletal elements within neurons. However, when tau becomes hyper-phosphorylated, it loses its activity leading to disruption of the cytoskeleton, causing synaptic transmission dysfunction and neuronal death (Alonso et al., 1994; Rajmohan and Reddy, 2015). Hyper-phosphorylated tau (P-tau) will form paired helical filaments, which will then aggregate to form the neurofibrillary tangles characteristic of AD. Studies with transgenic mouse models of AD suggest that Aβ toxicity is mediated by tau. It has also been shown that Aβ plays a role in triggering the hyper-phosphorylation of tau, suggesting that generation of Aβ precedes the accumulation of P-tau (Rapoport et al., 2002). Oxidative stress from other sources, such as decreased levels of insulin-like growth factor 1, has also been implicated in the formation of P-tau, leading to decreased cell viability. Loss of cytoskeletal integrity and subsequent neuronal death leads to the symptoms associated with AD.

#### Phosphorylated Tau Interaction With Drp1 and Defective Mitophagy

In a previous study, the Reddy lab (Manczak and Reddy, 2012a) tested whether P-tau interacted with Drp1 and attempted to elucidate how mitochondria are damaged in the progression of AD. They also investigated GTPase Drp1 enzymatic activity, which is critical for mitochondrial division in postmortem brain tissues from patients with AD as well as brain tissues from three different lines of transgenic APP, APP/PS1, and 3XTg.AD mice. Using co-immunoprecipitation and immunofluorescence analyses, they demonstrated the physical interaction between P-tau and Drp1 for the first time. Mitochondrial fission-linked GTPase Drp1 activity was significantly elevated in the postmortem frontal cortex tissues from AD patients and cortical tissues from APP, APP/PS1, and 3XTg.AD mice. Based on these findings, they concluded that Drp1 interacts with P-tau, likely leading to excessive mitochondrial fragmentation and mitochondrial synaptic deficiencies and ultimately leading to neuronal damage and cognitive decline (Manczak and Reddy, 2012a).

#### Phosphorylated Tau Interaction With Voltage-Dependent Anion Channel 1 Protein and Defective Mitophagy

To determine the role of mitochondrial outer membrane protein, VDAC1 and its interaction with p-tau in AD, the Reddy group (Manczak and Reddy, 2012b) studied the interaction between VDAC1 and phosphorylated tau, using cortical tissues from AD patients, control subjects, APP, APP/PS1, and 3XTg.AD mice. They found increased levels of VDAC1 in the cortical tissues from the brains of patients with AD, relative to control subjects. Co-immunoprecipitation and co-labeling analysis of postmortem AD brains, brain tissues from tau transgenic mice revealed that VDAC1 interacted with phosphorylated tau in the brains of AD patients and 3XTg.AD mice. They concluded that VDAC1 interacts with phosphorylated tau, which may, in turn, block mitochondrial pores, leading to mitochondrial dysfunction in AD pathogenesis.

Based on these observations, they propose that reduced levels of VDAC1 and phosphorylated tau may reduce the abnormal interaction between VDAC1 and phosphorylated tau. Reduced levels of VDAC1 and phosphorylated tau may maintain normal mitochondrial pore opening and pore closure, ultimately leading to normal mitochondrial function, mitochondria supplying ATP to nerve terminals, and boosting synaptic and cognitive function in AD (Manczak and Reddy, 2012b).

### Mitochondrial Dysfunction in Alzheimer’s Disease

Another common finding in AD postmortem brains is signs of oxidative damage and mitochondrial dysfunction (Reddy, 2007; Swerdlow et al., 2010). Mitochondria are responsible for the majority of ATP generation and produces ATP through the ETC, a series of complexes found within the inner mitochondrial membrane. However, many byproducts are produced during this process, including ROS, superoxide (O_2_^–^), hydroxyl radicals (OH.), and hydrogen peroxide (H_2_O_2_) (Reddy and Oliver, 2019). ROS production plays a role in the degradation of both chromosomal and mitochondrial DNA, leading to compromised production of machinery, which can lead to even further ROS production and cell death. Both Aβ and P-tau have been associated with mitochondrial dysfunction in some way as well.

In several studies, it has been shown that microtubule destabilization from overexpression of tau and hyper-phosphorylation of tau leads to disruption of cellular trafficking (Ebneth et al., 1998). Kinesin, the motor protein responsible for transport to the cell periphery, is preferentially inhibited by P-tau. In neurons where organelle transportation is important, it was observed that mitochondria would concentrate in the cell body and would not be present in neurites (Stamer et al., 2002). Other organelles would likewise be affected, including peroxisomes, which help to alleviate oxidative stress. The increased concentration of organelles in the cell body prevents further production of organelles leading to a decrease in the numbers of important organelles, including mitochondria and peroxisomes. With the deficit of these organelles, neurites are made vulnerable by the decreased production of energy and increased susceptibility to oxidative stress. Interruption of kinesin-driven transport also negatively impacts the ability of APP to be transported into axons and dendrites (Reddy et al., 2004). This leads to the accumulation of APP within cell bodies, which can then be processed into Aβ.

Many lines of research have shown that Aβ and APP play a more direct role in mitochondrial dysfunction. Research done on mice at various stages of AD showed that genes regulating mitochondrial metabolism and regulators of apoptosis were upregulated (Reddy et al., 2004). These findings suggest that energy metabolism in the presence of Aβ impairs mitochondrial energy metabolism and that the upregulation of genes is a compensatory response. The same study examined mRNA expression in patients with early AD and definite AD and found downregulation of mitochondrial genes in complex 1 of the ETC, whereas genes for complexes III and IV were upregulated in both populations. Increased expression of complexes III and IV suggests that greater demand is put on the mitochondria for energy output. Aβ also directly interfaces with mitochondrial proteins. *In vitro* studies show Aβ peptides (25-35) are capable of blocking the entry of proteins into mitochondria leading to mitochondrial dysfunction, membrane depolarization, increased ROS production, and altered mitochondrial morphology (Reddy and Beal, 2005; Moreira et al., 2007). Increased ROS production from mitochondria also activates the fission proteins Drp1 and Fis1, causing mitochondrial fragmentation (Barsoum et al., 2006). Studies have also shown that Aβ and APP can enter mitochondria, and Aβ is able to form oligomers within mitochondria (Devi et al., 2006; Manczak et al., 2006). To further reinforce the role of Aβ, genetic analysis of individuals with familial AD commonly show defects in amyloid-beta precursor protein (APP) and presenilin 1 and 2 (Martin-Maestro et al., 2016). Aberrant APP produces more Aβ-42, the isoform of Aβ that is implicated in pathology, and presenilin 1 and 2 are essential cofactors for γ-secretase, which is the final step of processing of APP into Aβ. Overall, the role of Aβ in mitochondrial disruption is multifaceted, with many different pathways in which Aβ can both, directly and indirectly, interfere with normal mitochondrial function.

## Dysfunctional Mitophagy in Aging

**FIGURE 4:**
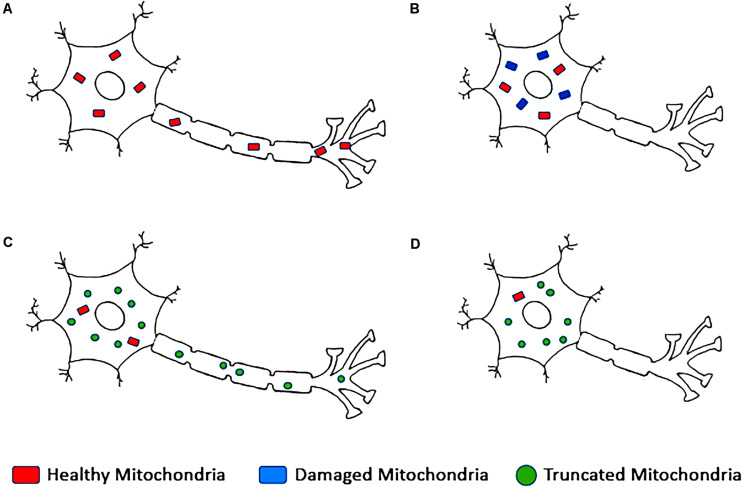
Mitochondrial profile of neurons in **(A)** healthy neurons, **(B)** neurons with disrupted microtubule networks, **(C)** neurons with upregulated mitochondrial dynamic proteins, and **(D)** damaged neurons observed in Alzheimer’s disease.

The major role of mitochondria in energy generation and ROS regulation makes their integrity paramount to cell health. Dysfunction in the mitochondrial health checkpoints and mitophagy machinery has been implicated in the acceleration of physiological aging and neurological diseases such as AD (Magrané et al., 2014; Ye et al., 2015; Rodolfo et al., 2018; Pradeepkiran and Reddy, 2020). The unique structure of neurons and their high energy demand makes neurons particularly reliant on proper mitochondrial function, and loss of mitochondrial integrity can lead to neuronal population loss and neurodegeneration (Figure 4). Neurons are also a non-proliferating cell type and so will accumulate cellular and oxidative stress over long periods (Bakthavachalam and Shanmugam, 2017).

As previously discussed, impairment of mitophagy can occur in several ways, but all ultimately lead to improper turnover of damaged mitochondria, accumulation of mitochondrial debris, and ultimately cell death (Figure 4). Neurodegenerative diseases have a complex set of symptoms and causative events, but most have familial forms that have helped to elucidate potential mechanisms underlying pathology. Several aberrant genes identified in these diseases have been linked to autophagy, implicating the role of autophagy in symptom genesis and progression. However, due to the complexity of the autophagic pathways and their regulators, the exact steps leading to pathology have been obfuscated. Despite the complexity, autophagy is generally broken down into a few steps: initiation, elongation, cargo recognition, and fusion with lysosomes.

## Defective Mitophagy in Alzheimer’s Disease

As previously discussed, both soluble Aβ and abnormally phosphorylated tau characteristically found in AD directly interact with mitochondria and impair function. Beyond the role Aβ plays in mitochondrial function, Aβ impacts mitochondrial mRNA and protein expression in mice with increased expression of APP (Reddy et al., 2018). Mitochondrial structural genes, autophagy genes, and mitophagy-specific genes have all been shown to change in some way. Of the mitochondrial structural genes, Drp1 and Fis1, which both play a role in mitochondrial fission, have increased expression. Mfn1, Mfn2, and OPA1, proteins of mitochondrial fusion, were shown to have decreased levels of mRNA expression. Combined, these alterations in protein expression lead to increased mitochondrial fission and fragmentation making it harder for the mitophagy machinery to keep up with the cellular demand to clean out damaged mitochondria. Other genes examined in this study included genes for proteins that regulated autophagy and mitophagy, which were all downregulated. Of note, PINK1 was observed to have a 2.4-fold decrease in mRNA expression, and the PINK1/Parkin pathway has been considered one of the main pathways in which mitophagy is carried out. These data suggest that the initiation and cargo recognition component of mitophagy is greatly inhibited by Aβ. Furthermore, initial Aβ accumulation and related mitochondrial damage aggressively induce the PINK1/Parkin pathway of mitophagy (Cai and Jeong, 2020). As the disease progresses, cytosolic Parkin is depleted, leading to reduced cellular mitophagy capabilities over time. Although this phenomenon does not inhibit the mitophagy pathway, it still decreases the cell’s capability to recycle damaged mitochondria leading to cellular stress. Studies have shown that basal levels of mitophagy can be restored in some cases when Parkin levels are overexpressed in mice (Martin-Maestro et al., 2016).

Early studies into the effects of abnormal tau on mitochondrial dynamics have focused primarily on the impairment of cellular trafficking. The destabilization of microtubule networks and interruption of organelle migration leads to the accumulation of damaged organelles within the neuronal soma. It has been found that tau also plays a role in inducing mitophagy by modulating membrane potential and Parkin levels (Hu et al., 2016). In individuals with increased total levels of tau and AD, increased mtDNA for mitophagy markers was observed, suggesting a mitophagy deficit within cells. The study also found that the membrane potential of mitochondria actually increases in the presence of abnormal tau, leading to decreased levels of PINK1 within mitochondria and subsequently decreased Parkin localization to mitochondria. Tau also directly interacts with Parkin, which directly interacts with the projection domain of Tau, leading to the cytosolic sequestration of Parkin (Cummins et al., 2019). Tau has also been shown to interact with Drp1, suggesting that tau also plays a role in the excessive mitochondrial fragmentation observed in AD (Manczak and Reddy, 2012a). As noted, the effects on mitochondrial dynamics by tau are widespread and inhibit mitophagy in multiple ways.

### Defective PINK1 and Parkin in Alzheimer’s Disease

Recently, Ye et al. (2015) studied Parkin-mediated mitophagy using mutant hAPP neurons and AD patient brains. They found Parkin-mediated mitophagy is involved in mutant hAPP neurons and postmortem AD brains. In the absence of Δψm dissipation reagents, hAPP neurons exhibit increased recruitment of cytosolic Parkin to depolarized mitochondria. Under AD-linked pathophysiological conditions, Parkin translocation predominantly occurs in the somatodendritic regions leading to decreased anterograde and increased retrograde mitochondrial axonal transport. Enhanced mitophagy was further confirmed in AD brains, accompanied by depletion of cytosolic Parkin over disease progression. Thus, aberrant accumulation of dysfunctional mitochondria in AD-affected neurons is likely attributable to inadequate mitophagy capacity and inability to clear damaged mitochondria. Altogether, these studies substantiate AD-linked chronic mitochondrial stress under *in vitro* and *in vivo* pathophysiological conditions.

Reddy et al. (2018) investigated the toxic effects of hippocampal mutant APP (mAPP) and Aβ in primary mouse hippocampal neurons (HT22) that express human APP Swedish mutation. Using quantitative reverse-transcriptase polymerase chain reaction, Western blotting and immunofluorescence, and transmission electron microscopy studies, they assessed mRNA and protein levels of synaptic, autophagy, mitophagy, mitochondrial dynamics, and biogenesis of proteins and assessed mitochondrial changes in mAPP-HT22 cells. Mitochondrial function was assessed by measuring the levels of hydrogen peroxide, lipid peroxidation, cytochrome c oxidase activity, and mitochondrial adenosine triphosphate. Increased levels of mRNA and protein levels of mitochondrial fission genes (Drp1 and Fis1) and decreased levels fusion (Mfn1, Mfn2, and Opa1) biogenesis (PGC1α, NRF1, NRF2, and TFAM), autophagy (ATG5 and LC3BI, LC3BII), mitophagy (PINK1 and TERT, BCL2 and BNIPBL), synaptic (synaptophysin and PSD95), and dendritic (MAP2) genes were found in mAPP-HT22 cells relative to WT-HT22 cells. Cell survival was significantly reduced by mAPP-HT22 cells. GTPase-Drp1 enzymatic activity was increased in mAPP-HT22 cells. Transmission electron microscopy revealed significantly increased mitochondrial numbers and reduced mitochondrial length in mAPP-HT22 cells. These findings suggest that hippocampal accumulation of mAPP and Aβ is responsible for the abnormal mitochondrial dynamics and defective biogenesis of MAP2, autophagy, mitophagy, and synaptic proteins as well as reduced dendritic spines and mitochondrial structural changes in mAPP hippocampal cells.

Reddy Lab (Manczak et al., 2018) also investigated the toxic effects of hippocampal mutant APP and Aβ in 12-month-old APP transgenic mice (Tg2576 strain). Using rotarod and Morris water maze tests, immunoblotting and immunofluorescence, Golgi-cox staining, and transmission electron microscopy, they assessed cognitive behavior, protein levels of synaptic, autophagy, mitophagy, mitochondrial dynamics, biogenesis, and dendritic protein MAP2 and also quantified dendritic spines and mitochondrial number and length in APP mice that express Swedish mutation. Mitochondrial function was assessed by measuring the levels of hydrogen peroxide, lipid peroxidation, cytochrome c oxidase activity, and mitochondrial ATP. Morris water maze and rotarod tests revealed that hippocampal and memory and motor learning and coordination were impaired in APP mice relative to WT mice. Increased levels of mitochondrial fission proteins and decreased levels of fusion, biogenesis, autophagy, mitophagy, synaptic, and dendritic proteins were found in 12-month-old APP mice relative to age-matched non-transgenic WT mice. Golgi-cox staining analysis revealed that dendritic spines were significantly reduced. Transmission electron microscopy revealed significantly increased mitochondrial numbers and reduced mitochondrial length in APP mice. These findings suggest that hippocampal accumulation of mutant APP and Aβ is responsible for abnormal mitochondrial dynamics and defective biogenesis, autophagy, mitophagy, and synaptic proteins and reduced dendritic spines and hippocampal-based learning and memory impairments in APP mice.

Fang et al. (2019b) also studied mitophagy in the progression of AD in pluripotent stem cell-derived human AD neurons, in animal AD models, and Aβ and tau *Caenorhabditis elegans* models of AD. They also found mitophagy is impaired in the hippocampus of AD patients, in induced pluripotent stem cell-derived human AD neurons, and in animal AD models. In both Aβ and tau *C. elegans* models of AD, mitophagy enhancers reversed memory impairment through PINK- 1-, Parkinson’s disease-related-1; parkin-, or DAF-16/FOXO-controlled germline-tumor affecting-1-dependent pathways. Mitophagy diminishes insoluble Aβ_1__–__42_ and Aβ_1__–__40_ and prevents cognitive impairment in an APP/PS1 mouse model through microglial phagocytosis of Aβ and suppression of neuroinflammation. Mitophagy enhancement abolishes AD-related tau hyperphosphorylation in human neuronal cells and reverses memory impairment in transgenic tau nematodes and mice. Their findings further support the findings of previous studies of Ye et al. (2015), Reddy et al. (2018), and Manczak et al. (2018) that defective mitophagy is a major cellular change in AD progression and pathogenesis.

**FIGURE 5:**
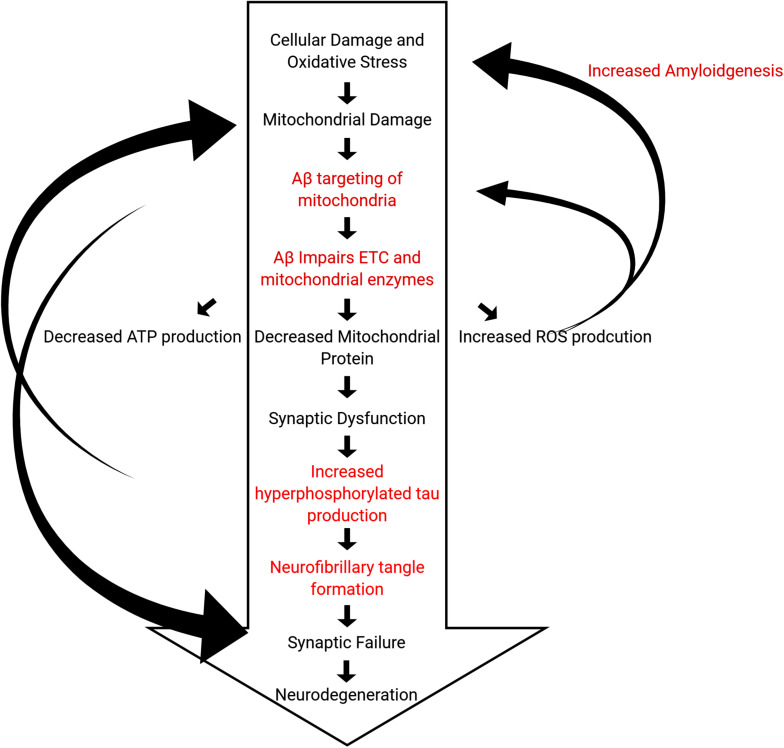
Hypothetical outline of events of the pathogenic steps in the mitochondrial cascade hypothesis leading to death of neurons. Steps outlined in black are observed in normal physiological aging, whereas steps outlined in red are characteristics of AD that accelerate these processes.

Overall, these studies clearly demonstrate that PINK1 and Parkin pathways are involved in AD. Both mRNA and protein levels of PINK1 and Parkin and other mitophagy and autophagy proteins are reduced in human and mouse AD cells and both APP and tau transgenic mouse models. These reductions inhibit/reduce mitochondrial function. Furthermore, the ability to clear damaged mitochondria through mitophagy is also compromised. The baseline-decrease in mitochondrial function associated with age initiates events leading to the formation and accumulation of Aβ, which exacerbates mitochondrial duress in what is termed the “mitochondrial cascade hypothesis,” introduced by Swerdlow et al. (2010) in Swerdlow et al. (2010) (Figure 5). The intricate interplay among Aβ, tau, and mitochondrial proteins is not completely understood, and further investigations into the events triggering symptoms of AD still need to be done.

## Conclusion and Future Directions

Mitochondria play a key role in the production of energy and balance of ROS within cells. Mitophagy, the selective breakdown and clearance of aberrant and dead mitochondria, is a regulatory process essential to promoting cellular health and maintaining healthy mitochondrial populations. As a person age, oxidative stress and cellular damage accumulate, and autophagic pathways can become overwhelmed. This is especially true in non-actively dividing cells such as neurons, and cortical degeneration is commonly observed in aging populations.

AD, a characteristic illness of aging, is associated with cognitive deficits, including loss of memory formation and increased loss of cortical mass. Furthermore, characteristic conglomerates of Aβ and fibrillary tangles of abnormally phosphorylated tau are observed within the brains of AD patients. Recent research also revealed that phosphorylated tau and fibrillary tangles are definitive AD features, both clinically and pathologically. Synaptic damage and defective mitophagy are early changes in disease progression, and as discussed earlier, aging plays a key role in synaptic and autophagy and mitophagy in AD progression and pathogenesis. Improved understanding of microglial activation and mitochondrial damage in neurons, particularly at synapses, is urgently needed.

In the past 20 years, the toxicity of these substrates has been studied extensively, and their role in neuronal death partially elucidated. The buildup of abnormal mitochondria is noted in AD neurons. More recently, studies have focused on the interaction between Aβ and tau on the components of mitophagy. Although some interactions between Aβ and tau and also Aβ and tau interactions with mitochondrial proteins and the components of mitophagy have been noted; the exact mechanisms and sequence of events leading to the genesis of AD have yet to be elucidated. Accumulation of damaged mitochondria, excessive mitochondrial fission, the buildup of ROS within cells, and compromised cellular health are all noted within neuronal populations in AD brains.

A major challenge in studies on the pathology of AD is identifying individuals with early-onset AD as the symptoms mimic what is normally expected in aging populations. Identification of the early events of AD within these populations can help elucidate the development of biomarkers and pathology in AD and outline the mechanisms by which symptoms occur. Further research could potentially develop mitophagy-based therapies to block or even reverse the adverse effects of AD. Further research is still needed to identify the role of mitophagy in AD.

## Author Contributions

PR contributed to the conceptualization and formatting of the article. MT and PR are responsible for writing, original draft preparation, and finalization of the manuscript. PR is responsible for funding acquisition. Both authors contributed to the article and approved the submitted version.

## Conflict of Interest

The authors declare that the research was conducted in the absence of any commercial or financial relationships that could be construed as a potential conflict of interest.

## Abbreviations

A β: Amyloid beta
AD: Alzheimer’s disease
BNIP3: BCL2/adenovirus E1B 19 kDa protein-interacting protein 3
Drp1: Dynamin-1-like protein
EF: Helix-loop-helix structural domain
ERMCS: ER mitochondria encounter structures
ETC: Electron transport chain
Fis1: Fission 1 protein
FUNDC1: FUN14 domain containing 1
LC3: Microtubule-associated protein 1A/1B-light chain 3
LRRK2: Leucine-rich repeat kinase 2
mAPP: Mutant amyloid beta precursor protein
Mff: Mitochondrial fission factor
Mfn1: Mitofusin-1
Mfn2: Mitofusin-2
Mid49: Mitochondrial dynamics protein MID49
Mid51: Mitochondrial dynamics protein MID51
Miro: Mitochondrial Rho GTPase
mtDNA: Mitochondrial DNA
NRF1: Nuclear respiratory factor 1
NRF2: Nuclear respiratory factor 2
NSF: N-ethylmaleimide sensitive factor
OMM: Outer membrane of mitochondria
Opa1: optic atrophy 1
OPTN: Optineurin
PGC-1 α: Peroxisome proliferator-activated receptor gamma coactivator 1-alpha
PINK1: PTEN-induced kinase 1
ROS: Reactive Oxygen Species
SIAH1: E3 ubiquitin-protein ligase SIAH1
TBK1: TANK-binding kinase 1
TEM: Transmission electron microscopy
TFAM: transcription factor A, mitochondrial.

## References

[B1] Ali M. Devkota S. Roh J. I. Lee J. Lee H. W. (2016). Telomerase reverse transcriptase induces basal and amino acid starvationinduced autophagy through mTORC1. *Biochem. Biophys. Res. Commun.* 478 1198–1204. 10.1016/j.bbrc.2016.08.094 27545609

[B2] Alonso A. C. Zaidi T. Grundke-Iqbal I. Iqbal K. (1994). Role of abnormally phosphorylated tau in the breakdown of microtubules in Alzheimer disease. *Proc. Natl. Acad. Sci. U.S.A.* 91 5562–5566. 10.1073/pnas.91.12.5562 8202528 PMC44036

[B3] Aman Y. Frank J. Lautrup S. H. Matysek A. Niu Z. Yang G. (2020). The NAD^+^-mitophagy axis in healthy longevity and in artificial intelligence-based clinical applications. *Mech. Ageing Dev.* 185:111194. 10.1016/j.mad.2019.111194 31812486 PMC7545219

[B4] Andreux P. A. Blanco-Bose W. Ryu D. Burdet F. Ibberson M. Aebischer P. (2019). The mitophagy activator urolithin A is safe and induces a molecular signature of improved mitochondrial and cellular health in humans. *Nat. Metab.* 1 595–603. 10.1038/s42255-019-0073-4 32694802

[B5] Aoki H. Iwado E. Eller M. S. Kondo Y. Fujiwara K. Li G. Z. (2007). Telomere 3’ overhang-specific DNA oligonucleotides induce autophagy in malignant glioma cells. *FASEB J.* 21 2918–2930. 10.1096/fj.06-6941com 17449721

[B6] Ashrafi G. Schwarz T. L. (2013). The pathways of mitophagy for quality control and clearance of mitochondria. *Cell Death Diff.* 20 31–42. 10.1038/cdd.2012.81 22743996 PMC3524633

[B7] Babbar M. Basu S. Yang B. Croteau D. L. Bohr V. A. (2020). Mitophagy and DNA damage signaling in human aging. *Mech. Ageing Dev.* 186:111207. 10.1016/j.mad.2020.111207 31923475 PMC7047626

[B8] Bakthavachalam P. Shanmugam P. S. T. (2017). Mitochondrial dysfunction – Silent killer in cerebral ischemia. *J. Neurol. Sci.* 375 417–423. 10.1016/j.jns.2017.02.043 28320180

[B9] Bakula D. Scheibye-Knudsen M. (2020). Mitophaging: mitophagy in aging and disease. *Front. Cell. Dev. Biol.* 15:239. 10.3389/fcell.2020.00239 32373609 PMC7179682

[B10] Barsoum M. J. Yuan H. Gerencser A. A. Liot G. Kushnareva Y. Graber S. (2006). Nitric oxide-induced mitochondrial fission is regulated by dynamin-related GTPases in neurons. *EMBO J.* 25 3900–3911. 10.1038/sj.emboj.7601253 16874299 PMC1553198

[B11] Blackburn E. H. Greider C. W. Szostak J. W. (2006). Telomeres and telomesrase: the path from maize, Tetrahymena and yeast to human cancer and aging. *Nature* 12 1133–1138. 10.1038/nm1006-1133 17024208

[B12] Cai Q. Jeong Y. Y. (2020). Mitophagy in Alzheimer’s disease and other age-related neurodegenerative diseases. *Cells* 9:150. 10.3390/cells9010150 31936292 PMC7017092

[B13] Castellazzi M. Patergnani S. Donadio M. Giorgi C. Bonora M. Bosi C. (2019). Autophagy and mitophagy biomarkers are reduced in sera of patients with Alzheimer’s disease and mild cognitive impairment. *Sci. Rep.* 9:20009. 10.1038/s41598-019-56614-5 31882960 PMC6934625

[B14] Chen G. Kroemer G. Kepp O. (2020). Mitophagy: an emerging role in aging and age-associated diseases. *Front. Cell. Dev. Biol.* 26:200. 10.3389/fcell.2020.00200 32274386 PMC7113588

[B15] Cheon S. Y. Kim H. Rubinsztein D. C. Lee J. E. (2019). Autophagy, cellular aging and age-related human diseases. *Exp. Neurobiol.* 28 643–657. 10.5607/en.2019.28.6.643 31902153 PMC6946111

[B16] Cuervo A. M. Wong E. (2014). Chaperone-mediated autophagy: roles in disease and aging. *Cell Res.* 24 92–104. 10.1038/cr.2013.153 24281265 PMC3879702

[B17] Cummins N. Tweedie A. Zuryn S. Bertran-Gonzalez J. Jurgen G. (2019). Disease-associated tau impairs mitophagy by inhibiting Parkin translocation to mitochondria. *EMBO J.* 38:99360. 10.15252/embj.201899360 30538104 PMC6356067

[B18] D’Amico D. Mottis A. Potenza F. Sorrentino V. Li H. Romani M. (2019). The RNA-binding protein PUM2 impairs mitochondrial dynamics and mitophagy during aging. *Mol. Cell.* 73 775–787. 10.1016/j.molcel.2018.11.034 30642763 PMC6396316

[B19] Deter R. L. De Duve C. (1967). Influence of glucagon, an inducer of cellular autophagy, on some physical properties of rat liver lysosomes. *J. Cell. Biol.* 33 437–449. 10.1083/jcb.33.2.437 4292315 PMC2108350

[B20] Devi L. Prabhu B. M. Galati D. F. Avadhani N. G. Anadartheerthavarada H. K. (2006). Accumulation of amyloid precursor protein in the mitochondrial import channels of human Alzheimer’s disease brain is associated with mitochondrial dysfunction. *J. Neurosci.* 26 9057–9068. 10.1523/JNEUROSCI.1469-06.2006 16943564 PMC6675337

[B21] Dice J. F. (2007). Chaperone-mediated autophagy. *Autophagy* 3 295–299. 10.4161/auto.4144 17404494

[B22] Ding W. Yin X. (2012). Mitophagy: mechanisms, pathophysiological roles, and analysis. *Biol. Chem.* 393 547–564. 10.1515/hsz-2012-0119 22944659 PMC3630798

[B23] Ding W. X. Ni H. M. Li M. Liao Y. Chen X. Stolz D. B. (2010). Nix is critical to two dinstinct phases of mitophagy, reactive oxygen species-mediated autophagy induction and Parkin-ubiquitin-p62-mediated mitochondrial priming. *J. Biol. Chem.* 285 27879–27890. 10.1074/jbc.M110.119537 20573959 PMC2934655

[B24] Dunn W. A. Jr. Cregg J. M. Kiel J. A. K. W. Ida J. K. Oku M. Yasuyoshi S. (2012). Pexophagy: the selective autophagy of peroxisomes. *Autophagy* 1 75–83. 10.4161/auto.1.2.1737 16874024

[B25] Ebneth A. Godemann K. Illenberger S. Trinczek B. Mandelkow E. M. Mandelkow E. (1998). Overexpression of tau protein inhibits kinesin-dependent trafficking of vesicles, mitochondria, and endoplasmic reticulum: implications for Alzheimer’s disease. *J. Cell. Biol.* 143 777–794. 10.1083/jcb.143.3.777 9813097 PMC2148132

[B26] Fang E. F. Hou Y. Lautrup S. Jensen M. B. Yang B. SenGupta T. (2019a). NAD^+^ augmentation restores mitophagy and limits accelerated aging in Werner syndrome. *Nat. Commun.* 10:5284. 10.1038/s41467-019-13172-8 31754102 PMC6872719

[B27] Fang E. F. Hou Y. Palikaras K. Adriaanse B. A. Kerr J. S. Yang B. (2019b). Mitophagy inhibits amyloid-β and tau pathology and reverses cognitive deficits in models of Alzheimer’s disease. *Nat. Neurosci.* 22 401–412. 10.1038/s41593-018-0332-9 30742114 PMC6693625

[B28] Fraga M. F. Esteller M. (2007). Epigenetics and aging: the targets and the marks. *Trends Genet.* 23 413–418. 10.1016/j.tig.2007.05.008 17559965

[B29] Glick D. Barth S. Macleod K. F. (2010). Autophagy: cellular and molecular mechanisms. *J. Pathol.* 221 3–12. 10.1002/path.2697 20225336 PMC2990190

[B30] Goiran T. Duplan E. Rouland L. Manaa W. Lauritzen I. Dunys J. (2018). Nuclear p53-mediated repression of autophagy involves PINK1 transcriptional down-regulation. *Cell Death Differ.* 25 873–884. 10.1038/s41418-017-0016-0 29352272 PMC5943347

[B31] Green D. R. Galluzzi L. Kroemer G. (2011). Mitochondria and the autophagy-inflammation-cell death axis in organismal aging. *Science* 333 1109–1112. 10.1126/science.1201940 21868666 PMC3405151

[B32] Greer E. L. Maures T. J. Hauswirth A. G. Green E. M. Leeman D. S. Maro G. S. (2010). Members of the H3K4 trimethylation complex regulate lifespan in a germline-dependent manner in *C. elegans*. *Nature* 466 383–387. 10.1038/nature09195 20555324 PMC3075006

[B33] Hale A. N. Ledbetter D. J. Gawriluk T. R. Rucker E. B. III (2012). Autophagy Regulation and role in development. *Autophagy* 9 951–972. 10.4161/auto.24273 24121596 PMC3722331

[B34] Hamley I. W. (2012). The amyloid beta peptide: a chemist’s perspective. role in Alzheimer’s and fibrillization. *Chem. Rev.* 112 5147–5192. 10.1021/cr3000994 22813427

[B35] Han S. Jeong Y. Y. Sheshadri P. Su X. Cai Q. (2020). Mitophagy regulates integrity of mitochondria at synapses and is critical for synaptic maintenance. *EMBO Rep.* 21:e49801 10.15252/embr.201949801 PMC7507095 32627320

[B36] Harper J. W. Ordureau A. Heo J. M. (2018). Building and decoding ubiquitin chains for mitophagy. *Nat. Rev. Mol. Cell. Biol.* 19 93–108. 10.1038/nrm.2017.129 29358684

[B37] Hayflick L. Moorhead P. S. (1961). The serial cultivation of human diploid cell strains. *Exp. Cell Res.* 25 585–621. 10.1016/0014-4827(61)90192-6 13905658

[B38] He C. Klionsky D. J. (2010). Regulation mechanisms and signaling pathways of autophagy. *Annu. Rev. Genet.* 43 67–93. 10.1146/annurev-genet-102808-114910 19653858 PMC2831538

[B39] Hou Y. Dan X. Babbar M. Wei Y. Hasselbalch S. G. Croteau D. L. (2019). Ageing as a risk factor for neurodegenerative disease. *Nat. Rev. Neurol.* 15 565–581. 10.1038/s41582-019-0244-7 31501588

[B40] Hu Y. Li X. C. Wang Z. H. (2016). Tau accumulation impairs mitophagy via increasing mitochondrial membrane potential and reducing mitochondrial Parkin. *Oncotarget* 7 17356–13468. 10.18632/oncotarget.7861 26943044 PMC4951217

[B41] Hyman B. T. Phelps C. H. Beach T. G. Bigio E. H. Cairns N. J. Carrillo M. C. (2012). National Institute on Aging-Alzheimer’s Association guidelines for the neuropathologic assessment of Alzheimer’s disease. *Alzheimers Dement.* 8 1–13. 10.1016/j.jalz.2011.10.007 22265587 PMC3266529

[B42] Kanki T. Wang K. Cao Y. Baba M. Klionsky D. J. (2009). Atg32 is a mitochondrial protein that confers selectivity during mitophagy. *Dev. Cell.* 17 98–109. 10.1016/j.devcel.2009.06.014 19619495 PMC2746076

[B43] Khang R. Zeh H. J. Lotze M. T. Tang D. (2011). The Beclin 1 network regulates autophagy and apoptosis. *Cell Death Differ.* 18 571–580. 10.1038/cdd.2010.191 21311563 PMC3131912

[B44] Kiffin R. Kaushik S. Zeng M. Bandyopadhyay U. Zhang C. Massey A. C. (2007). Altered dynamics of the lysosomal receptor for chaperone-mediated autophagy with age. *J. Cell. Sci.* 120 782–791. 10.1242/jcs.001073 17284523

[B45] Lauritzen I. Pardossi-Piquard R. Bourgeois A. Becot A. Checler F. (2019). Does intraneuronal accumulation of carboxyl-terminal fragments of the amyloid precursor protein trigger early neurotoxicity in Alzheimer’s disease? *Curr. Alzheimer Res.* 16 453–457. 10.2174/1567205016666190325092841 30907322

[B46] Laurtizen I. Pardossi-Piquard R. Bourgeois A. Pagnotta S. Biferi M. G. Barkats M. (2016). Intraneuronal aggregation of the β-CTF fragment of APP (C99) induces Aβ-independent lysosomal-autophagy pathology. *J. Biol. Chem.* 293 15419–15428. 10.1074/jbc.R118.003999 27138984 PMC4947121

[B47] Lett O. (2016). Ambra1 in autophagy and apoptosis: implications for cell survival and chemotherapy resistance. *Oncol. Lett.* 12 367–374. 10.3892/ol.2016.4644 27347152 PMC4906955

[B48] Li W. W. Li J. Bao J. K. (2012). Microautophagy: lesser-known self-eating. *Cell. Mol.* 69 1125–1136. 10.1007/s00018-011-0865-5 22080117 PMC11114512

[B49] Li X. Huang L. Lan J. Feng X. Li P. Wu L. (2020). Molecular mechanisms of mitophagy and its roles in neurodegenerative diseases. *Pharmacol. Res.* 11:105240. 10.1016/j.phrs.2020.105240 33053441

[B50] Liang W. J. Gustafsson ÅB. (2020). The aging heart: mitophagy at the center of rejuvenation. *Front. Cardiovasc. Med.* 7:18 10.3389/fcvm.2020.00018 PMC7042393 32140472

[B51] Lopez-Otin C. Blasco M. A. Partridge L. Serrano M. Kroemer G. (2013). The hallmarks of aging. *Cell* 153 1194–1217. 10.1016/j.cell.2013.05.039 23746838 PMC3836174

[B52] Lou G. Palikaras K. Lautrup S. Scheibye-Knudsen M. Tavernarakis N. Fang E. F. (2020). Mitophagy and neuroprotection. *Trends Mol. Med.* 26 8–20. 10.1016/j.molmed.2019.07.002 31375365

[B53] Luo H. Zhang R. Krigman J. McAdams A. Ozgen S. Sun N. A. (2020). Healthy heart and a healthy brain: looking at mitophagy. *Front. Cell. Dev. Biol.* 6:294. 10.3389/fcell.2020.00294 32435642 PMC7218083

[B54] Lynch-Day M. A. Klionsky D. J. (2010). The Cvt pathway as a model for selective autophagy. *FEBS Lett.* 584 1359–1366. 10.1016/j.febslet.2010.02.013 20146925 PMC2843786

[B55] Maegawa S. Hinkal G. Kim H. S. Shen L. Zhang L. Zhang J. (2010). Widespread and tissue specific age-related DNA methylation changes in mice. *Genome Res.* 20 332–340. 10.1101/gr.096826.109 20107151 PMC2840983

[B56] Magrané J. Cortez C. Gan W. B. Manfredi G. (2014). Abnormal mitochondrial transport and morphology are common pathological denominators in SOD1 and TDP43 ALS mouse models. *Hum. Mol. Genet.* 23 1413–1424. 10.1093/hmg/ddt528 24154542 PMC3929084

[B57] Majeski A. E. Dice J. F. (2004). Mechanisms of chaperone-mediate autophagy. *Int. J. Biochem. Cell. Biol.* 36 2435–2444. 10.1016/j.biocel.2004.02.013 15325583

[B58] Manczak M. Anekonda T. S. Henson E. Park B. S. Quinn J. Reddy P. H. (2006). Mitochondria are a direst site of Aβ accumulation in Alzheimer’s disease neurons: implications for free radical generation and oxidative damage in disease progression. *Hum. Mol. Genet.* 15 1437–1449. 10.1093/hmg/ddl066 16551656

[B59] Manczak M. Calkins M. J. Reddy P. H. (2011). Impaired mitochondrial dynamics and abnormal interaction of amyloid beta with mitochondrial protein Drp1 in neurons from patients with Alzheimer’s disease: implications for neuronal damage. *Hum. Mol. Genet.* 20 2495–2509. 10.1093/hmg/ddr139 21459773 PMC3109997

[B60] Manczak M. Kandimalla R. Yin X. Reddy P. H. (2018). Hippocampal mutant APP and amyloid beta-induced cognitive decline, dendritic spine loss, defective autophagy, mitophagy. *Hum. Mol. Genet.* 27 1332–1342. 10.1093/hmg/ddy042 29408999 PMC6455948

[B61] Manczak M. Reddy P. H. (2012a). Abnormal interaction between the mitochondrial fission protein Drp1 and hyperphosphorylated tau in Alzheimer’s disease neurons: implications for mitochondrial dysfunction and neuronal damage. *Hum. Mol. Gen.* 21 2538–2547. 10.1093/hmg/dds072 22367970 PMC3349426

[B62] Manczak M. Reddy P. H. (2012b). Abnormal interaction of VDAC1 with amyloid beta and phosphorylated tau causes mitochondrial dysfunction in Alzheimer’s disease. *Hum. Mol. Genet.* 21 5131–5146. 10.1093/hmg/dds360 22926141 PMC3490521

[B63] Maria Fimia G. Stoykova A. Romagnoli A. Giunta L. Di Bartolomeo S. Nardacci R. (2007). Ambra1 regulates autophagy and development of the nervous system. *Nature* 447 1121–1125. 10.1038/nature05925 17589504

[B64] Markaki M. Tavernarakis N. (2020). Mitochondrial turnover and homeostasis in ageing and neurodegeneration. *FEBS Lett.* 594 2370–2379. 10.1002/1873-3468.13802 32350855

[B65] Martín-Maestro P. Gargini R. García E. Simón D. Avila J. García-Escudero V. (2019). Mitophagy failure in APP and tau overexpression model of Alzheimer’s disease. *J. Alzheimers Dis.* 70 525–540. 10.3233/JAD-190086 31256128

[B66] Martin-Maestro P. Gargini R. Perry G. Avila J. Garcia-Escudero V. (2016). Park2 enhancement is able to compensate mitophagy alterations found in sporadic Alzheimer’s disease. *Hum. Mol. Genet.* 25 792–806. 10.1093/hmg/ddv616 26721933 PMC4743695

[B67] Mehrpour M. Esclatine A. Beau I. Codogno P. (2010). Overview of macroautophagy regulation in mammalian cells. *Cell Res.* 20 748–762. 10.1038/cr.2010.82 20548331

[B68] Mijaljica D. Prescott M. Devenish R. J. (2011). Microautophagy in mammalian cells: revisiting a 40-year-old conundrum. *Autophagy* 7 673–682. 10.4161/auto.7.7.14733 21646866

[B69] Moreira P. I. Santos M. S. Oliveira C. R. (2007). Alzheimer’s disease: a lesson from mitochondrial dysfunction. *Antioxid. Redox. Signal.* 9 1621–1630. 10.1089/ars.2007.1703 17678440

[B70] Moskalev A. A. Shaposhnikov M. V. Plyusnina E. N. Zhavoronkov A. Budovsky A. Yanai H. (2013). The role of DNA damage and repair in aging through the prism of Koch-like criteria. *Ageing Res. Rev.* 12 661–684. 10.1016/j.arr.2012.02.001 22353384

[B71] Murphy M. P. Levine H. (2010). Alzheimer’s disease and the β-amyloid peptide. *J. Alzheimers Dis.* 19:311. 10.3233/JAD-2010-1221 20061647 PMC2813509

[B72] Nassour J. Radford R. Correia A. Fusté J. M. Schoell B. Jauch A. (2019). Autophagic cell death restricts chromosomal instability during replicative crisis. *Nature* 565 659–663. 10.1038/s41586-019-0885-0 30675059 PMC6557118

[B73] Ney P. A. (2015). Mitochondrial autophagy: origins, significance, and role of BNIP3 and NIX. *Biochim. Biophs. Acta* 1853 2775–2783. 10.1016/j.bbamcr.2015.02.022 25753537

[B74] Oh J. Youn C. K. Jun Y. Jo E. R. Cho S. I. (2020). Reduced mitophagy in the cochlea of aged C57BL/6J mice. *Exp. Gerontol.* 137:110946. 10.1016/j.exger.2020.110946 32387126

[B75] Okamoto K. Kondo-Okamoto N. Ohsumi Y. (2009). Mitochondria-anchored receptor Atg32 mediates degradation of mitochondria via selective autophagy. *Dev. Cell.* 17 98–109. 10.1016/j.devcel.2009.06.013 19619494

[B76] Oku M. Sakai Y. (2018). Three distinct types of microautophagy based on membrane dynamics and molecular machineries. *Bioessays* 40 42–48. 10.1002/bies.201800008 29708272

[B77] Oliver D. M. A. Reddy P. H. (2019a). Dynamics of dynamin-related protein 1 in Alzheimer’s disease and other neurodegenerative diseases. *Cells* 8:961. 10.3390/cells8090961 31450774 PMC6769467

[B78] Oliver D. M. A. Reddy P. H. (2019b). Molecular basis of Alzheimer’s disease: focus on mitochondria. *J. Alzheimers Dis.* 72 S95–S116. 10.3233/JAD-190048 30932888

[B79] Pakpian N. Phopin K. Kitidee K. Govitrapong P. Wongchitrat P. (2020). Alterations in mitochondrial dynamic-related genes in the peripheral blood of Alzheimer’s disease patients. *Curr. Alzheimer Res.* 17 616–625. 10.2174/1567205017666201006162538 33023448

[B80] Palikaras K. Lionaki E. Tavernarakis N. (2015). Coordination of mitophagy and mitochondrial biogenesis during ageing in *C. elegans*. *Nature* 521 525–528. 10.1038/nature14300 25896323

[B81] Palikaras K. Lionaki E. Tavernarakis N. (2018). Mechanisms of mitophagy in cellular homeostasis, physiology and pathology. *Nat. Cell. Biol.* 20 1013–1022. 10.1038/s41556-018-0176-2 30154567

[B82] Parzych K. R. Klionsky D. J. (2014). An overview of autophagy: morphology, mechanism, and regulation. *Antioxid. Redox Signal.* 20 460–473. 10.1089/ars.2013.5371 23725295 PMC3894687

[B83] Pickrell A. M. Youle R. J. (2015). The roles of PINK1, PARKIN, and mitchondrial fidelity in Parkinson’s disease. *Neuron* 85 257–273. 10.1016/j.neuron.2014.12.007 25611507 PMC4764997

[B84] Pradeepkiran J. A. Reddy P. H. (2020). Defective mitophagy in Alzheimer’s disease. *Ageing Res. Rev.* 64:101191. 10.1016/j.arr.2020.101191 33022416 PMC7710581

[B85] Quinsay M. N. Thomas R. L. Lee Y. Gustafsson A. B. (2010). Bnip3-mediated mitochondrial autophagy is independent of the mitochondrial permeability transition pore. *Autophagy* 6 855–862. 10.4161/auto.6.7.13005 20668412 PMC3039735

[B86] Rajmohan R. Reddy P. H. (2015). Amyloid-beta and phosphorylated tau accumulations cause abnormalities at synapses of Alzheimer’s disease neurons. *J. Alzheimers Dis.* 57 975–999. 10.3233/JAD-160612 27567878 PMC5793225

[B87] Rapoport M. Dawson H. N. Ferreira A. (2002). Tau is essential to β-amyloid-induced neurotoxicity. *Proc. Natl. Acad. Sci. U.S.A.* 99 6364–6369. 10.1073/pnas.092136199 11959919 PMC122954

[B88] Reddy P. H. (2006). Mitochondrial oxidative damage in aging and Alzheimer’s disease: implications for mitochondrially targeted antioxidant therapeutics. *J. Biolmed. Biotechnol.* 3:31372. 10.1155/JBB/2006/31372 17047303 PMC1559913

[B89] Reddy P. H. (2007). Mitochondrial dysfunction in aging and Alzheimer’s disease: strategies to protect neurons. *Antioxid Redox Signal* 9 1647–1658. 10.1089/ars.2007.1754 17696767

[B90] Reddy P. H. (2008). Mitochondrial medicine for aging and neurodegenerative diseases. *Neuromol. Med.* 10 291–315. 10.1007/s12017-008-8044-z 18566920 PMC3235551

[B91] Reddy P. H. Beal M. F. (2005). Are mitochondria critical in pathogenesis of Alzheimer’s disease? *Brain Res. Rev.* 49 618–632. 10.1016/j.brainresrev.2005.03.004 16269322

[B92] Reddy P. H. Beal M. F. (2008). Amyloid beta, mitochondrial dysfunction and synaptic damage: implications for cognitive decline in aging and Alzheimer’s disease. *Trends Mol. Med.* 14 45–53. 10.1016/j.molmed.2007.12.002 18218341 PMC3107703

[B93] Reddy P. H. McWeeney S. Park B. S. Manczak M. Gutala R. V. Partovi D. (2004). Gene expression profiles of transcripts in amyloid precursor protein transgenic mice: up-regulation of mitochondrial metabolism and apoptotic genes is an early cellular change in Alzheimer’s disease. *Hum. Mol. Genet.* 13 1225–1240. 10.1093/hmg/ddh140 15115763

[B94] Reddy P. H. Oliver D. M. (2019). Amyloid beta and phosphorylated tau-induced defective autophagy and mitophagy in Alzheimer’s disease. *Cells*. 8:488. 10.3390/cells8050488 31121890 PMC6562604

[B95] Reddy P. H. Reddy T. P. (2011). Mitochondria as a therapeutic target for aging and neurodegenerative diseases. *Curr. Alzheimer Res.* 8 393–409. 10.2174/156720511795745401 21470101 PMC3295247

[B96] Reddy P. H. Williams J. Smith F. Bhatti J. S. Kumar S. Vijayan M. (2017). MicroRNAs, aging, cellular senescence, and Alzheimer’s disease. *Prog. Mol. Biol. Transl Sci.* 146 127–171. 10.1016/bs.pmbts.2016.12.009 28253983

[B97] Reddy P. H. Yin X. Manczak M. Kumar S. Pradeepkiran J. A. Vijayen M. (2018). Mutant APP and amyloid beta-induced defective autophagy, mitophagy, mitochondrial structural and functional changes and synaptic damage in hippocampal neurons from Alzheimer’s disease. *Hum. Mol. Gen.* 27 2502–2516. 10.1093/hmg/ddy154 29701781 PMC6031001

[B98] Reiss A. B. Arain H. A. Stecker M. M. Siegart N. M. Kasselman L. J. (2018). Amyloid toxicity in Alzheimer’s disease. *Rev. Neurosci.* 29 613–627. 10.1515/revneuro-2017-0063 29447116

[B99] Rodolfo C. Campello S. Cecconi F. (2018). Mitophagy in neurodegenerative disease. *Neurochem. Int.* 117 156–166. 10.1016/j.neuint.2017.08.004 28797885

[B100] Rossi D. J. Bryder D. Seita J. Nussenzweig A. Hoejimakers J. Weissman I. L. (2007). Deficiencies in DNA damage repair limit the function of haematopoietic stem cells with age. *Nature* 447 725–729. 10.1038/nature05862 17554309

[B101] Rubinsztein D. C. Marino G. Kroemer G. (2011). Autophagy and Aging. *Cell* 146 682–695. 10.1016/j.cell.2011.07.030 21884931

[B102] Schiavi A. Maglioni S. Palikaras K. Shaik A. Strappazzon F. Brinkmann V. (2015). Iron-starvation-induced mitophagy mediates lifespan extension upon mitochondrial stress in *C. elegans*. *Curr. Biol.* 25 1810–1822. 10.1016/j.cub.2015.05.059 26144971

[B103] Selkoe D. J. (2010). Alzheimer’s disease: genes, proteins, and therapy. *Physiol. Rev.* 81 741–766. 10.1152/physrev.2001.81.2.741 11274343

[B104] Shammas M. A. (2011). Telomeres, lifestyle, cancer, and aging. *Curr. Opin. Clin. Nutr. Metab. Care* 14 28–34. 10.1097/MCO.0b013e32834121b1 21102320 PMC3370421

[B105] Shaw A. C. Joshi S. Greenwood H. Panda A. Lord J. M. (2010). Aging of the innate immune system. *Curr. Opin. Immunol.* 22 507–513. 10.1016/j.coi.2010.05.003 20667703 PMC4034446

[B106] Shefa U. Jeong N. Y. Song I. O. Chung H. J. Kim D. Jung J. (2019). Mitophagy links oxidative stress conditions and neurodegenerative diseases. *Neural Regen. Res.* 14 749–756. 10.4103/1673-5374.249218 30688256 PMC6375051

[B107] Sienko K. Poormassalehgoo A. Yamada K. Goto-Yamada S. (2020). Microautophagy in plants: consideration of its molecular mechanism. *Cells* 9:887 10.3390/cells9040887 PMC7226842 32260410

[B108] Stamer K. Thies E. Mandeljow E. Mandelkow E. M. (2002). Tau blocks traffic of organelles, neurofilaments, and APP vesicles in neurons and enhances oxidative stress. *J. Cell. Biol.* 156 1051–1063. 10.1083/jcb.200108057 11901170 PMC2173473

[B109] Sun N. Youle R. J. Finkel T. (2016). The mitochondrial basis of aging. *Mol. Cell.* 61 654–666. 10.1016/j.molcel.2016.01.028 26942670 PMC4779179

[B110] Swerdlow R. H. Burns J. M. Khan S. M. (2010). The Alzheimer’s disease mitochondrial cascade hypothesis. *J. Alzheimers Dis.* 20 265–279. 10.3233/JAD-2010-100339 20442494 PMC2883665

[B111] Tal R. Winger G. Ecker N. Klionsky D. J. Abeliovich H. (2007). Aup1p, a yeast mitochondrial protein phosphatase homolog, is required for efficient stationary phase mitophagy and cell survival. *J. Biol. Chem.* 282 5617–5624. 10.1074/jbc.M605940200 17166847

[B112] Varghese N. Werner S. Grimm A. Eckert A. (2020). Dietary mitophagy enhancer: a strategy for healthy brain aging? *Antioxidants* 9:E932. 10.3390/antiox9100932 33003315 PMC7600282

[B113] Vellai T. (2009). Autophagy genes and ageing. *Cell Death Differ.* 16 94–102. 10.1038/cdd.2008.126 19079287

[B114] Wang Z. T. Lu M. H. Zhang Y. Ji W. L. Lei L. Wang W. (2019). Disrupted-in-schizophrenia-1 protects synaptic plasticity in a transgenic mouse model of Alzheimer’s disease as a mitophagy receptor. *Aging Cell* 18:e12860. 10.1111/acel.12860 30488644 PMC6351828

[B115] Yang X. Zhang M. Dai Y. Sun Y. Aman Y. Xu Y. (2020). Spermidine inhibits neurodegeneration and delays aging via the PINK1-PDR1-dependent mitophagy pathway in *C. elegans*. *Aging* 12 16852–16866. 10.18632/aging.103578 32902411 PMC7521492

[B116] Ye X. Sun X. Starovoytov V. Cai Q. (2015). Parkin-mediated mitophagy in mutant hAPP neurons and Alzheimer’s disease patient brains. *Hum. Mol. Genet.* 24 2938–2951. 10.1093/hmg/ddv056 25678552 PMC4406302

[B117] Yoo S. Jung Y. A. (2018). Molecular approach to mitophagy and mitochondrial dynamics. *Mol. Cells* 41 18–26. 10.14348/molcells.2018.2277 29370689 PMC5792708

[B118] Youle R. J. Narendra D. P. (2011). Mechanisms of mitophagy. *Nat. Rev. Mol. Cell Biol.* 12 9–14. 10.1038/nrm3028 21179058 PMC4780047

[B119] Zaffagnini G. Martens S. (2016). Mechanisms of selective autophagy. *J. Mol. Biol.* 428 1714–1724. 10.1016/j.jmb.2016.02.004 26876603 PMC4871809

[B120] Zhang J. Ney P. A. (2009). Role of BNIP3 and NIX in cell death, autophagy, and mitophagy. *Cell Death Differ.* 16 939–946. 10.1038/cdd.2009.16 19229244 PMC2768230

[B121] Zhang Y. W. Thompson R. Zhang H. Xu H. (2011). APP processing in Alzheimer’s disease. *Mol. Brain* 4:3. 10.1186/1756-6606-4-3 21214928 PMC3022812

